# Silencing women’s sexuality: global AIDS policies and the case of the female condom

**DOI:** 10.7448/IAS.16.1.18452

**Published:** 2013-07-08

**Authors:** Anny JTP Peters, Francien TM van Driel, Willy HM Jansen

**Affiliations:** 1 Institute for Gender Studies, Radboud University Nijmegen, Nijmegen, The Netherlands; 2 Centre for International Development Issues Nijmegen, Radboud University Nijmegen, Nijmegen, The Netherlands; 3 Rutgers WPF, Dutch International Expert Centre on Sexuality, Utrecht, The Netherlands

**Keywords:** global policy, AIDS prevention, gender, sexual agency, female condom

## Abstract

**Introduction:**

The female condom is the only evidence-based AIDS prevention technology that has been designed for the female body; yet, most women do not have access to it. This is remarkable since women constitute the majority of all HIV-positive people living in sub-Saharan Africa, and gender inequality is seen as a driving force of the AIDS epidemic. In this study, we analyze how major actors in the AIDS prevention field frame the AIDS problem, in particular the female condom in comparison to other prevention technologies, in their discourse and policy formulations. Our aim is to gain insight into the discursive power mechanisms that underlie the thinking about AIDS prevention and women’s sexual agency.

**Methods:**

We analyze the AIDS policies of 16 agencies that constitute the most influential actors in the global response to AIDS. Our study unravels the discursive power of these global AIDS policy actors, when promoting and making choices between AIDS prevention technologies. We conducted both a quantitative and qualitative analysis of how the global AIDS epidemic is being addressed by them, in framing the AIDS problem, labelling of different categories of people for targeting AIDS prevention programmes and in gender marking of AIDS prevention technologies.

**Results:**

We found that global AIDS policy actors frame the AIDS problem predominantly in the context of gender and reproductive health, rather than that of sexuality and sexual rights. Men’s sexual agency is treated differently from women’s sexual agency. An example of such differentiation and of gender marking is shown by contrasting the framing and labelling of male circumcision as an intervention aimed at the prevention of HIV with that of the female condom.

**Conclusions:**

The gender-stereotyped global AIDS policy discourse negates women’s agency in sexuality and their sexual rights. This could be an important factor in limiting the scale-up of female condom programmes and hampering universal access to female condoms.

## Introduction

Central in the formulation of global AIDS policies is the creation of access to the existing evidence-based HIV prevention technologies [1]. Condoms are proven to be effective HIV prevention technologies, and male condoms are widely accepted and easily accessible, although less so within marriage as they are mainly associated with AIDS prevention in extra-marital sexual relations [2]. Female condoms, however, are far less accepted and accessible, and have remained expensive and highly underfunded [3]. Consequently, most women and girls in sub-Saharan Africa lack access to the female condom [4]. This is despite the fact that women and girls make up 61% of all HIV-positive people living in sub-Saharan Africa [5]. Gender inequality is considered one of the key drivers of the sub-Saharan AIDS epidemic [6]. Nonetheless, it is not clear how global AIDS policy actors include the concept of gender in their programming, especially when prioritizing HIV prevention technologies.

Gender, which generally denotes the social and cultural constructions of femininity and masculinity, is multi-dimensional and works out differently among different cultural contexts. Feminist theories and women’s health research share the common intention of reflecting critically on biology as a stable and fixed framework. Gender produces diversity. Some consider gender as “a set of practices that bring reproductive distinctions between human bodies into social processes” [7]. It is therefore more than the cultural inscription of meaning on a pre-given sex and concerns the discursive means by which “a natural sex” is produced [8]. Similarly, gender designates the very apparatus of production whereby the sexes themselves are established [9]. We have to bear in mind that “our beliefs about gender affect what kinds of knowledge scientists produce about sex in the first place” [10, p. 3]. The concept of gender includes the power relations that produce and perpetuate gender identities [11]. Therefore, a gender analysis concerns an analysis of power and is increasingly apprehensive with the ways in which other structures of inequality and power intersect with those of gender, such as sexuality [12], race and ethnicity [13], and religion [14].

Global AIDS policies, similar to other policies [15], result from a complex configuration of interests of a range of actors; the formulation of such policies is not a neutral process. Gender arrangements, which are shaped by and shape individual actions, are an intrinsic part of policy formulation processes. Thus, particular ways of thinking about gender and about AIDS gain ascendancy, and determine the frame through which the AIDS problem and its solutions are formulated and adopted.

Framing is a necessary, nonetheless often implicit dimension of policy formulation [16]. It is defined as the way policy actors perceive and interpret the features of the phenomenon at hand, and how they attach meaning to it. The global AIDS problem can be framed in many ways: as a health, medical, pharmaceutical, economic, social, sexual, moral, political, security and/or development problem [17,18]. Its framing by policy makers reveals how they choose to view the AIDS problem. This framing is a continuous process, influenced by a variety of stakeholders who bring in their particular positions, perceptions and solutions. Power positions come into play, and eventually certain views will prevail and become the dominant way of thinking, while other views will be overshadowed [19]. We consider AIDS policies part of what Foucault would call “bio power,” a tool by which people’s sexuality can be administered, cultivated and controlled. Therefore, policy papers are considered as one important discursive practice that shape perceptions of sexuality [20].

In this article, we study how global AIDS policy actors frame the AIDS problem in relation to women’s sexuality and the female condom by analyzing the discourse used in policy papers. It is beyond the scope of this article to prove the impact of this discourse on female condom use. As the female condom is a technology used during sexual intercourse, and as AIDS concerns a sexually transmitted disease that affects people’s sexual and reproductive health, we choose to analyze how the AIDS problem is being framed by global AIDS actors in the context of sexuality. Sexuality is a central aspect of humanity, encompassed by gender ideologies. Both gender and sexuality are culturally constructed and key to consider when addressing the AIDS problem [21–24].

The World Health Organisation (WHO) defines sexual health as a state of physical, emotional, mental and social wellbeing related to sexuality. Sexual health requires a positive and respectful approach to sexuality and sexual relationships, as well as the possibility of having pleasurable and safe sexual experiences, free of coercion, discrimination and violence. For sexual health to be attained and maintained in a population, the sexual rights of all persons must be respected, protected and fulfilled [25]. This approach of sexual health includes the notion of sexual agency. Globally, sexual and reproductive health is recognized as universal concepts since the International Conference on Population and Development (ICPD) in 1994. While the initial draft Programme of Action that was formulated at the ICPD included sexual rights in a broad sense for both men and women, the text that was eventually adopted restricted women’s rights to decisions about human reproduction [26]. Similarly, women’s reproductive rights are often referred to in the policies of individual agencies, rather than their sexual rights [27,28]. The WHO’s working definition of sexual rights is the right of all persons, free of coercion, discrimination and violence, to the highest attainable standard of sexual health, including access to sexual and reproductive healthcare services; the right to information related to sexuality; to sexuality education; to respect for bodily integrity; to partner choice; to the decision whether or not to be sexually active; to consensual sexual relations; to consensual marriage; to the decision whether or not and when to have children; and to a satisfying, safe and pleasurable sexual life [29]. Remarkable is the fact WHO states that this definition of sexual rights “does not represent an official WHO position, and should not be used or quoted as WHO definition.” Certain political and religious leaders, especially the more conservative ones, consider sexuality to solely serve reproductive interests and they deny men’s and women’s entitlement to experience and enjoy sexuality independent of reproduction. In this study, we critically look at the way the global AIDS policy actors label people and deal with the notion of sexual agency when framing the AIDS problem.

Labelling people by classifying them into target groups with specific characteristics is a common practice in development cooperation, and global AIDS programmes are no exception [30]. AIDS policy makers construct categories of people to target their programmes and to allocate and manage their resources. Categorization of target groups is typically based on epidemiological risk profiles. In many countries in sub-Saharan Africa women have an epidemiological profile that is based on the label “being married” [31] and this high risk is associated with their husbands’ extra-marital sexual activities [32,33]. Categorizing according to risk profiles is a common practice and may look efficient, but it is neither value free nor necessarily a one-way process [34].

As labelling determines access to resources and services, certain population groups organize themselves around a perceived common identity and seek to make themselves visible in society, in particular towards the state, service providers and aid agencies. Groups of people may use an established label to influence global AIDS policy actors. The gay movement in the western world [35], and the sex workers movement in Kolkata, India [36], for example successfully organized themselves in an effort to reduce HIV transmission through campaigns and lobbying, which were instrumental to making themselves visible and being heard by politicians. In this sense, labelling is positively related to social activism, as it helps particular interest groups to create solidarity and mobilize resources for a common purpose [37].

But labelling has its downsides too. Being pointed out as a high-risk group or victim in global AIDS policies, may lead to stigmatization and exclusion. HIV-positive people often experience stigma, discrimination and blame [38,39]. And among them, those that are poor and female in general suffer disproportionately [40–42]. Stigma comes in different forms, but it is basically an attribute that is deeply discrediting within a particular social setting [43]. Moreover, Deacon et al. [44] argue that negative labelling or stigmatization, often turns into self-stigmatization, due to feelings of helplessness and powerlessness. Stigmatization thus can influence people’s agency and their confidence to claim their rights, including access to social services and the use of (AIDS) prevention technologies [45].

AIDS policy actors are in a position to mark some technologies as more appropriate than others, and to leave certain solutions as unmarked. We are interested in the gender marking of health technologies and the effect this may have on people’s perceptions and acts. The word “condom” for instance is an unmarked term as far as gender is concerned: it is a device that is designed for the male body. As with many other supposedly gender neutral terms, however – think of youth criminals, politicians or house-keepers – most people automatically associate these with one of the two sexes. Yet, by not gender marking a term, this common sense association goes unnoticed. Other words, however, are typically marked in gender terms; for example, the female condom and male circumcision. Gender marking is generally applied to situations or technologies in which the normative and so-called non-normative categories are hierarchically positioned. The gender unmarked sense often remains politically unnoticed, such as “condom,” a term that is often used to designate the male condom. The marked sense, on the other hand, is more heavily articulated and observed [46].

Our study primarily aims to unravel the discursive power of global AIDS policy actors in depicting women’s sexuality and sexual rights and in prioritizing specific AIDS prevention technologies for specific categories of people, in particular the female condom. To assess this power and the way it works out in policy papers of global AIDS actors, we use the concepts of framing, labelling and gender marking. Basic to our approach is seeing policies not only as “data,” but also as “discourse.” “Data” because policies give information on the AIDS problem, population groups and proposed solutions; and “discourse” because policy makers employ a specific terminology and narrative strategies when defining their target groups and stating their objectives, which may in turn affect the persons involved and their attitudes and acceptance of certain prevention technologies [47]. In analyzing these discourses, the terminology, how often certain terms are used, and their various underlying meanings, are important. So are the forms and the events during which the discourse is presented [3]. Foucauldian research from a relativist epistemological perspective defines “discourse” as a group of statements, objects or events that represent knowledge about, or construct a particular topic. Therefore, “discourse analysis” is an analysis of the ways in which knowledge is created through the existing discourses; the question of which discourse prevails and whose interest it serves are most important [48].

We seek an answer to the question what the discursive power of global AIDS policy actors has been in dealing with women’s sexuality and sexual rights, and in particular the female condom. This central question is divided into three sub-questions. What AIDS prevention technologies do global AIDS policy actors prioritize in their policy papers? How do they frame the AIDS problem in the context of gender and sexuality? How are categories of people labelled, and what are the consequences for the gender marking of AIDS prevention technologies? After presenting our methodology, we describe the results of our analysis in relation to these questions, followed by a discussion and conclusion.

## Methodology

This article examines the policies of 16 international AIDS actors that are most essential to the global AIDS governance structure [49]. As noted by Rushton, the literature is quite unanimous on the question who the key actors are in the global governance of AIDS [50]. Among the key actors, there are seven UN agencies, four bilateral agencies (three European and one American), the World Bank (WB), the European Union (EU), the Global Fund for AIDS Tuberculosis and Malaria (GFATM), the Bill and Melinda Gates Foundation (BMGF) and the Global HIV Prevention Working Group (GHPWG). The last one (i.e. GHPWG) is an international AIDS policy advisory panel, convened by BMGF and the Henry J Kaiser Family Foundation. We analyze the way these institutions have formulated their AIDS policies and strategic documents, specifically the attention they give to the different AIDS prevention technologies and how they link them to various target groups, each with their distinct labels. Table 1 presents the data sources of our study. All are available on the websites of these institutions. Of each actor, we selected the most recent and substantive policy or strategic document that used the words HIV or AIDS in the title. An exception was made for the strategic document of GFATM, which did not have HIV or AIDS in its title. When institutions had more than one policy or strategic document on HIV/AIDS on their website, we selected the one of which the title indicated the area of HIV prevention or the most recent one.

**Table 1:** Data source: title, year of publication and number of pages of 16 global AIDS policies or AIDS strategic documents

Agency	Title of AIDS policy or AIDS strategic document	Year	Pages
UN agencies			
UNGASS: United Nations General Assembly on AIDS [51]	Uniting for universal access towards zero new HIV infections, zero-discrimination, and zero AIDS related death.	2011	24
UNAIDS: Joint United Nations Programme on HIV/AIDS [52]	Getting to zero. UNAIDS 2011–2015 strategy.	2010	63
UNDP: United Nations Development Programme [53]	Leadership for results. UNDP’s response to HIV/AIDS.	2005	32
UNESCO: United Nations Educational, Scientific and Cultural Organization [54]	UNESCO’s strategy for HIV/AIDS.	2011	30
WHO: World Health Organization [55]	The global health sector strategy on HIV/AIDS 2011–2015.	2011	40
UNIFEM: United Nations Entity for Gender Equality and the Empowerment of Women [56]	Women: meeting the challenges of HIV/AIDS.	2005	8
UNICEF: United Nations Children Fund [57]	Opportunity in crisis. Preventing HIV from early adolescence to young adulthood.	2011	68
Bilateral development agencies			
DFID: British Department for International Development [58]	Achieving Universal Access. The UK’s strategy for halting and reversing the spread of HIV in the developing world.	2008	65
MoFA (Netherlands): Netherlands Ministry of Foreign Affairs [59]	Choices and opportunities. Policy memorandum HIV/AIDS and sexual and reproductive health and rights in foreign policy.	2009	54
SIDA: Swedish International Development Cooperation Agency [60]	Government, the right to a future: Policy for Sweden’s International HIV and AIDS efforts.	2009	26
PEPFAR (USA): President’s Emergency Plan for AIDS Relief [61]	Guidance for the prevention of sexually transmitted HIV infections.	2011	53
Global foundations and alliances			
GFATM: Global Fund for AIDS, Tuberculosis and Malaria [62]	The global fund strategy 2012 – 2016: investing for impact.	2011	22
WB: World Bank [63]	The World Bank’s commitment to HIV/AIDS in Africa. Our agenda for action, 2007–2011.	2008	58
EU: European Union [64]	A European programme for Action to confront HIV/AIDS, malaria and tuberculosis (2007–2011).	2005	17
BMGF: Bill and Melinda Gates Foundation [65]	The Gates Foundation’s HIV strategy.	2010	3*
GHPWG: Global HIV prevention working group [66]	Bringing HIV prevention to scale: an urgent global priority.	2007	30

* This strategy paper is short and does not include an analysis of the global AIDS problem, only solutions.

The year of appearance of the latest policy paper for each actor varies, but at the time of writing this article, all 16 AIDS policies are valid. On average, an AIDS policy paper consists of 37 pages, ranging from 3 to 68 pages. The purpose of our analysis is not to highlight differences between the agencies, but rather to detect commonalities in the way they prioritize AIDS prevention technologies, how they frame the AIDS problem in the context of gender and sexuality, and consequently the way they label people to target programmes and gender mark their technologies.

First, we listed all AIDS prevention technologies that the 16 actors refer to in their policies. We counted a total of nine different technologies and grouped them, as recommended by the Interagency Coalition on AIDS and Development, in the existing and potential technologies [67]. Five existing HIV prevention technologies are mentioned: two types of condoms, that is male condoms and female condoms; two anti-retroviral-based technologies (ART), that is prevention of mother-to-child HIV transmission (PMTCT) and test-and-treat; and male circumcision. Three potential HIV prevention technologies, all still in their trial phase, are mentioned: pre-exposure prophylaxis (PrEP), microbicides and vaccines. For the purpose of our article, we also included sexuality education, which is dubbed the “social vaccine” for HIV prevention [68].

Second, having identified the nine technologies, we counted the frequency with which each of them was mentioned. The frequency count gives insight into the relative importance and priority accorded to each technology by the 16 actors. From our epistemological perspective, digital frequency counts are important, as they reflect an underlying power to define what is important and at the same time produce certain views and assumptions [69]. As to the framing the AIDS problem in the context of gender and sexuality, we covered this by using the text analysis method to obtain frequency counts for four key terms: “gender,” “sexuality” (or “sexual behaviour”), “reproductive health” and “sexual rights” (including “sexual and reproductive health and rights” and its abbreviation “SRHR”). We used “gender” as a single term, despite its variety of understandings in different cultural contexts. We composed a second frequency table for the four key terms for framing the AIDS problem, and complemented this with qualitative information from the texts. We then analyzed the way people are categorized and labelled in the 16 AIDS policies. We listed all terms used and identified 14 different labels, as shown in Table 2.

**Table 2:** List of 14 labels identified, by sex

Categories	Labels
Females	Woman/women, mother(s)/maternal, girl(s), lesbian(s)/women who have sex with women/homosexual women
Males	Man/men, father(s)/paternal, boy(s), gay(s)/men who have sex with men/homosexual men
Undefined sex	Youth/young people, transgender/bisexual(s)/queer(s), sex worker(s), drug user(s), prisoner(s), migrant(s)

The labels and the frequency with which they are used can inform about global AIDS actors’ assumptions about the target groups of their policies [70]. We therefore composed a third frequency table for the 14 labels that categorize people. The qualitative data are presented as quotes taken from the 16 global AIDS policy papers.

To add further substance to the discourse on the female condom, we analyzed United Nations Population Fund (UNFPA) experiences on female condoms. UNFPA falls outside our 16 source documents, since it does not have a stand-alone AIDS policy. However, since UNFPA is the designated UN lead agency on SRHR, we analyzed their latest official document on female condoms as an extra source document [71]. An extra literature search was done to see whether the use of the generic term “condom” included the female condom.

## Results

### AIDS prevention technologies

Table 3 presents the frequency with which the nine AIDS prevention technologies appear in each of the 16 AIDS policy papers.

**Table 3:** Frequency of 9 AIDS prevention technologies in policy papers of 16 global AIDS policy actors

	Existing technologies					Potential technologies				
			
Name of the institute	Condom	Female condom	PMTCT	Test-and-treat	Male circumcision	Micro-bicides	Vaccine	PrEP	Sexuality education	Total
UNGASS	5	1	8	9	4	4	4	0	0	35
UNAIDS	5	1	14	14	5	2	3	0	6	50
UNDP	1	0	0	5	0	0	0	0	0	6
UNESCO	0	0	2	1	1	1	0	1	64	70
WHO	8	4	11	21	2	2	5	4	1	58
UNIFEM	7	3	0	2	0	1	0	0	0	13
UNICEF	87	5	16	50	20	4	1	2	28	213
DFID	30	5	13	1	8	15	6	1	0	79
Netherlands	10	6	3	3	2	5	6	1	6	42
SIDA	2	0	0	0	0	1	1	0	10	14
PEPFAR	89	19	17	47	44	4	2	20	2	244
GFATM	0	0	2	2	2	0	0	0	0	6
World Bank	17	2	13	9	2	3	1	0	0	47
EU	2	0	2	2	0	5	5	0	0	16
BMGF	0	0	2	1	4	4	11	7	0	29
GHPWG	60	0	17	40	40	1	3	1	0	162
Total	323	46	120	207	134	52	48	37	117	1084
Percentage	30%	4%	11%	19%	12%	5%	4%	3%	11%	100%
Ranking	1	8	3	2	4	6	7	9	5	
Number of agencies	13	9	13	15	12	14	12	8	7	16

The condom is the most frequently mentioned AIDS prevention technology (30% of more than a thousand times that specific AIDS prevention technologies are mentioned in the 16 source documents). They are mentioned by all but 3 of the 16 actors: the BMGF, the Global Fund and UNESCO. Condoms are in frequency followed by test-and-treat (19%) and PMTCT (11%). Both technologies are based on anti-retroviral treatment and together they represent 30% of the total number of times a technology is mentioned. Male circumcision comes fourth: it is mentioned by 12 agencies with a total frequency of 12%. Sexuality education is mentioned by seven agencies, with a total frequency of 11%, mainly in the policy papers of UNESCO and UNICEF. The majority of agencies (9 out of 16) do not mention sexuality education in their policies. The three technologies that are still in their trial phase are mentioned for a total of 12%, including microbicides (5%), vaccines (4%), and PrEP (3%).

Although the condom is mentioned by 13 actors with a total frequency of 30%, only nine agencies mention the female condom with a total frequency of 4%, most of which is accounted for by PEPFAR. Besides BMGF, who missed out on the condom, an additional six global AIDS policy actors (EU, GFATM, GWGHP, UNDP, UNESCO and SIDA) that mentioned the condom completely miss out on the female condom. It might be possible that some agencies group the female condom under the gender unmarked technology “condom.” To find out if people, and thus also likely global AIDS policies, refer to both male and female condoms when using the generic term condom, we studied 10 most recently published articles with condom(s) in the title in three journals: *AIDS*, *AIDS and Behavior*, and *AIDS Patient Care and STDs*; all articles published before July 2012. These 10 articles actually considered male condoms only when mentioning condoms. When articles do consider female condoms, they specifically mark them. This extra analysis demonstrates that female condoms are indeed generally excluded when the gender unmarked technology of condoms is addressed. This means that we can safely assume that when the gender unmarked term condom is used, it typically involves the male condom, and when female condoms are involved they are explicitly mentioned.

The limited attention to female condoms as one of the AIDS prevention technologies stands in sharp contrast to the multiple references made to other technologies. Two technologies, microbicides and vaccines, are still in the development stage and therefore still not proven effective to be widely implemented [72]. They are slightly more often mentioned than the female condom, which already was proven effective since 1993 [73]. Interestingly, all 16 global actors suggest investments in research and development for new technologies. For instance DFID states in its policy: “The UK will increase at least 50% of our funding for AIDS vaccines and microbicides research” [58, p. 58]. Simultaneously, the 16 global actors suggest the scaling-up of existing proven technologies. The GHPWG for example positions: “We could slow and even begin to reverse the trajectory of the global HIV epidemic by using the prevention tools currently at our disposal. To realize the promise of available HIV prevention tools, they must be brought to scale” [66, p. 61]. Female condoms are such a proven, simple and cost-effective prevention tool, and it is currently at our disposal. However, none of the global actors explicitly offers to scale up this technology. There are no phrases to be found in any of the policy papers that suggest any intention to scaling up the use of female condoms.

We conclude that some technologies of which the effectiveness is partial, such as male circumcision and microbicides, catch more attention in the number of times mentioned in the analyzed text (12%, respectively 5%) than the female condom (4%), which is proven effective. This suggests a bias in policy preference which is not supported by the available evidence on the efficacy of available technologies. The high recognition of the male condom, as an effective unmarked technology, compared to the low recognition of the female condom, as a marked technology, is noteworthy and solicits the question how the problem of AIDS is framed in the context of gender and sexuality.

## The framing of AIDS in the context of sexuality

Table 4 shows the frequency distribution of four key terms used for framing the AIDS problem in the context of gender and sexuality.

**Table 4:** Frequency of gender, reproductive health, sexuality and sexual rights in 16 global AIDS policy papers

Agency	Gender	Reproductive health	Sexuality	Sexual rights	Total
UNGASS	20	4	0	0	24
UNAIDS	62	17	17	5	101
UNDP	41	0	0	0	41
UNESCO	80	1	46	0	127
WHO	47	22	3	1	73
UNIFEM	16	2	1	0	19
UNICEF	17	26	19	1	63
DFID	32	20	8	18	78
Netherlands	13	45	14	22	94
SIDA	27	11	5	11	54
PEPFAR	28	6	20	0	54
GFATM	9	0	0	0	9
World Bank	34	24	4	0	62
EU	5	3	1	2	11
BMGF	0	0	0	0	0
GHPWG	4	7	7	0	18
Total	435	188	145	60	828
	53%	23%	18%	7%	100%
Rank	1	2	3	4	
Number of agencies	15	13	12	7	15

Of all four terms considered, the term “gender” is by far most often mentioned (53%). All 16 AIDS policies, except the one of the BMGF, somehow give importance to gender as illustrated by the following quotes. The UNDP policy paper states for example: “HIV/AIDS is not only about a virus. It is also about shame and guilt, gender inequality, power relations, silence and denial, stigma and discrimination” [63, p. 4]. The PEPFAR policy declares for instance: “Gender inequality is a cross-cutting issue: all PEPFAR prevention programmes must take gender dynamics into account in order to be effective” [61, p. 37]. Yet another illustration taken from the UNAIDS policy paper: “Scaling up effective gender-sensitive and gender-transformative interventions that engage men is needed just as much as efforts to ensure that women have roles in decision-making from the household level to the parliament. These must include programmes to reduce harmful gender norms by actively engaging men and boys” [52, p. 45].

Reproductive health (23%) is used more often than sexuality (18%). Global AIDS policy actors appear to favour the term gender instead of sexuality, when framing the AIDS problem. Sometimes they are connected. This is illustrated in the DFID policy paper: “Women and men face different risks and barriers in relation to the AIDS epidemic and in accessing services. Gender inequalities mean that women and girls cannot always decide if, when, how and with whom they have sex, or when to access basic services. Violence against women and girls significantly increases their risk of HIV infection. Women and girls report increased violence for refusing sex, requesting condom use, accessing HIV counselling and testing, and for testing HIV-positive. Women and girls also bear the greatest burden of care, including caring for orphans and those who are sick” [58, p. 24]. But the term sexuality, just like gender, neither does feature in the BMGF policy nor in that of UNGASS, UNDP or GFATM.

The term sexual rights is used even less (7%). Apart from the four actors who do not refer to sexuality in their policy papers, an additional five agencies omit the term “sexual rights”: UNESCO, UNIFEM, WB, PEPFAR and GHPWG. Seven agencies, predominantly the ones based in Europe, however, do use the term “sexual rights”: UNAIDS, WHO, UNICEF, EU, Great Britain, the Netherlands and Sweden. In this way, they refer to what was expressed during the ICPD, held in 1994 and re-enforced in 1999 during the 21st special session of the General Assembly of the United Nations:

We should assure women’s ability to control their own fertility. These rights rest on the recognition of the basic right of all couples and individuals to decide freely and responsibly the number, spacing and timing of their children and to have the information and means to do so, and the right to attain the highest standard of sexual and reproductive health. [74]

These seven actors proficiently use the discourse of SRHR in their AIDS policies. Especially the Europe based agencies see it as essential. The DFID policy, for example, states: “Expanded access to Sexual Reproductive Health and Rights (SRHR), including family planning, is a key part of an effective AIDS response. Successful HIV prevention is about enabling individuals, couples and communities to make healthy choices about personal aspects of their lives – particularly sexual behaviour” [58, p. 15]. Another example is found in the EU policy that states: “Political dialogue with countries is crucial to addressing and defending basic principles and to raising and discussing sensitive issues at the highest political level. At global level, the European Commission’s voice may be due to a formal mandate, e.g. in trade policy, or to the EC taking the initiative or being asked by EU Member States to take on such a role. This is sometimes also the case in UN processes, where it is the EU Member States that are fully represented and have a formal voice. Examples include preparations of UNGASS, follow-up to the MDGs, and UN conferences on gender equality and SRHR – all of which are intimately linked to the policy issues discussed in this Programme for Action” [64, p. 10]. The above quotes show that these European actors frame AIDS not only in the context of gender, sexuality, and reproductive rights, but also in the context of sexual rights. Typically we could not find any evidence of this type of framing of the AIDS problem in the AIDS policies of the Global Fund (GFTATM) and the WB. This is remarkable, in light of the fact that these two agencies derive a large part of their funding from European actors who consider sexual rights important.

We conclude that global AIDS policy actors frame AIDS predominantly in relation to gender and/or reproductive health, rather than to sexuality and sexual rights. Below we will show what this implies for the way people are labelled in AIDS policies.

## How people are labelled?

Table 5 shows which categories of people are mentioned by the 16 global AIDS policies and the frequencies with which the terms are used.

**Table 5:** Frequency with which 14 labels of people are mentioned by 16 global AIDS policy actors

	Females				Males				Sex not specified						
				
Agency	Women	Mothers	Girls	Lesbians	Men	Fathers	Boys	Gays	Sex workers	Drug users	Youth	Prisoners	Trans-gender	Migrants	Total
UNGASS	41	8	18	0	6	0	3	7	4	5	17	2	1	1	113
UNAIDS	68	14	27	0	25	0	3	25	38	19	32	5	16	1	273
UNDP	50	0	16	0	8	0	4	0	0	0	5	1	0	5	89
UNESCO	40	2	30	0	5	0	6	9	0	2	69	2	4	1	170
WHO	30	11	7	0	3	0	2	9	10	7	14	8	7	1	109
UNIFEM	48	7	12	0	10	1	4	0	0	0	2	0	0	0	84
UNICEF	81	22	49	0	35	6	17	31	30	54	276	1	1	0	603
DFID	68	13	19	1	13	1	6	22	23	13	26	13	3	5	226
Netherlands	58	3	10	0	3	0	0	7	9	17	34	5	0	2	148
SIDA	27	0	17	1	13	0	7	7	5	7	24	1	1	1	111
PEPFAR	64	8	10	0	32	0	2	10	6	1	52	1	1	0	187
GFATM	2	8	0	0	0	0	0	0	0	0	1	1	0	1	13
World Bank	42	7	7	0	6	0	0	8	9	4	38	1	0	0	122
EU	7	2	3	0	2	0	0	1	1	4	0	2	0	1	23
BMGF	0	2	0	0	0	0	0	0	1	0	0	0	0	0	3
GHPWG	48	17	4	0	0	0	1	5	36	42	32	10	0	0	195
Total	674	124	229	2	161	8	55	141	172	175	622	53	34	19	2469
	27%	5%	9%	0.1%	7%	0.3%	2%	6%	7%	7%	25%	2%	1%	1%	100%
Programme target		x						x	x	x	x				
Rank	1	8	3	14	6	13	9	7	5	4	2	10	11	12	
Number of agencies	15	14	14	2	13	3	11	12	12	12	14	14	7	10	

The 16 global AIDS policies use 14 different labels to differentiate people, which altogether were mentioned a total of 2469 times (=100%). Women (27%) are the most frequently mentioned category, much more often than men (7%). Girls (9%) get more attention than boys (2%), and mothers (5%) more than fathers (0.3%). For males, the label homosexual (or gay; 6%) is used almost as often as that of men (7%), while for females, the label homosexual (or lesbian; 0.1%) is hardly applied. It is mentioned by only two agencies, just once by each.

Of the 14 categories, the AIDS policies of nine agencies label five categories of people to target AIDS prevention programmes: youth (25%), sex workers (7%), drug users (7%), gays/men who have sex with men (6%) and mothers (5%) [51,52,55,57–61,66]. Two of these labels refer to one sex: gays, which are always men, and mothers, which are women by definition. The rest of these labels: youth, sex workers and drug users can be both women and men: they are sex unmarked. We have argued earlier that unmarked categories leave a lot of space for “common sense” assumptions that can lead to neglect of one gender (e.g. sex workers always being assumed females or drugs users as males). Another observation is that there is overlap between these labelled categories.

AIDS policies also categorize people as women (27%), girls (9%), men (7%), boys (2%), fathers (0.3%) and lesbians (0.1%), but these labels are not used for targeting purposes in AIDS prevention programmes [52, p. 9]. Women and girls are specifically mentioned to emphasize the severity of the global AIDS epidemic. For example, the AIDS policy paper of the WB states: “We are more conscious that this horrific scourge has disproportionately hit women and young girls” [63, p. 11]. In addition, women and girls are extensively referred to in order to justify investments in new technologies. For example the policy of DFID proposes to “increase by at least 50% funding for research and development of AIDS vaccines and microbicides over 2008 to 2013, to reduce the impact of the disease on women and girls” [58, p. 5]. Another illustration is the statement in the policy of the Ministry of Foreign Affairs of the Netherlands, where categories women and girls are used to portray them as victims, who are coerced by males: “Millions of girls are forced into their first sexual experience, some through violence. Women in long term relationships run the risk of violence if they press their partner to use a condom” [59, p. 12].

The discourse on men and boys differs from the one on women and girls. Men are described as highly sexually active, not wanting to use condoms and not taking responsibility for their sexual acts, such as stated in the PEPFAR policy paper: “Males are resistant to the use of condoms” [61, p. 18]. Another example is in the policy of the WB: “The principal elements in the reduction of HIV transmission include a decrease in the number of partners among adults – particularly highly sexually active men” [63, p. 14]. Representing men as active transmitters of HIV, resistant to HIV prevention technologies, is often part of describing the AIDS problem in sub-Saharan Africa [75]. It indicates that AIDS policy makers, like all people in their daily social interactions, are “doing gender,” meaning that – in words and acts, and by being held accountable for it – they express and construct dominant norms of masculinity and femininity [76]. Through their specific constructions of masculinity and femininity, policy makers not only define who receives which type of support, but also influence the availability of effective technologies by which people can protect themselves against HIV/AIDS infection [77].

While AIDS policies do differentiate between the sexes by labelling women and men, the category of young people is predominantly left gender unmarked in AIDS prevention. For example, the UNGASS document declares that “young people are leading the global prevention revolution” [51, p. 5] , suggesting that girls and boys are alike and face similar challenges. Another example comes from the policy paper of UNAIDS: “It is critical that we empower and facilitate young people as change agents in activating their communities to redress harmful social norms governing sexuality, gender roles and other behaviour” [52, p. 35]. In the policy of WHO, youth is described as a category of people who lack information: “Young people must have access to education on sex and sexuality to ensure they have comprehensive, correct knowledge about HIV; currently it remains low” [55, p. 19]. We highlight the fact that the label youth is largely left gender unmarked in AIDS policies, while the norms on young people’s sexual behaviour and their gender roles are known to be different, in all cultural settings [78]. Young people’s sexual behaviour is a sensitive issue in global politics [78]. By not gender marking young people in global AIDS policies, global AIDS policy actors silence gender, an essential aspect of sexuality, especially for young people on their way towards adulthood. By choosing not to challenge gender differences in sexual behaviour, taboos surrounding young people’s sexuality are not being problematized and hence they persist without being addressed.

We conclude that labelling is a common practice in global AIDS policies in order to target specific population groups. On the one hand it leads to exclusion of certain categories of people from AIDS prevention programmes and technologies; on the other hand it focuses the attention to other groups, with the inherent risk of stigmatizing them. Moreover, the labels that are being used imply different notions of sexual agency for specific groups of people. Before we draw further conclusions, we investigate gender labelling more in-depth, by considering two typically gender marked AIDS prevention technologies more closely: the female condom and male circumcision.

## Gender marked AIDS prevention technologies

Male circumcision (12%) is mentioned three times more often as a prevention technology in global AIDS policies than the female condom (4%; see Table 3). Twelve of the 16 global AIDS agencies refer to male circumcision in their policies. The remaining four actors (UNDP, UNIFEM, EU, and SIDA) had their AIDS policies approved between 2005 and 2009, when male circumcision was not yet widely promoted. Most agencies promote a package of interventions, with male circumcision as an integrated intervention. The WHO policy, for example, states: “Interventions to reduce sexual transmission include behaviour change counselling, male and female condom programming, early initiation of antiretroviral therapy, safe male circumcision (in high HIV-prevalence settings), post-exposure prophylaxis, and quality-assured HIV testing and counselling of sero-discordant couples” [55, p. 11]. Some policies warn that male circumcision does not provide full protection; for example the paper of the GHPWP, which states: “Health experts stress the importance of accompanying the roll-out of adult male circumcision with strengthened HIV prevention efforts to avoid giving circumcized men the impression that the procedure obviates the need for other standard prevention precautions, such as condom use or limiting the number of sexual partners” [66, p. 10]. Others, for example BMGF, see male circumcision as a technology that needs to be promoted: “The foundation is investing in advocacy efforts to encourage more rapid scale-up of male circumcision for HIV prevention” [65, p. 2]. We saw this type of advocacy during the 2010 International AIDS Conference in Vienna. Bill Gates showed the promotional film documentary: “Reducing HIV risk through circumcision.” It did not indicate in any way that circumcized men still need to use a condom to practice safer sex. Neither did it pay any attention to women, who may be at greater risk if their male partners believe they are fully protected once they are circumcized. Some researchers argue that male circumcision was being promoted in a rather absolute manner, like a “magic bullet” [79,80]. It appears as though some global AIDS policies promote male circumcision as a panacea for HIV prevention, at least in countries with a high HIV prevalence. However, many ethical issues are not resolved [81], and adequate education about HIV infection risk and human rights before men undergo circumcision are still lacking [82].

Meanwhile, female condom programmes receive much less support. The global AIDS policies are generally positive about the promotion of condoms, both male and female condoms. The WHO policy document states: “HIV programmes should promote equity between the sexes in sexual decision-making, including negotiation of safer sex and use of male and female condoms” [55, p. 28]. And according to PEPFAR: “A growing body of evidence shows that effective female condom promotion to both women and men can increase the proportion of protected sex acts” [61, p. 17]. UNIFEM writes: “The female condom provides women with an option where they may have greater control in negotiating condom use” [56, p. 4]. The female condom is actually a technology that potentially gives women more control over their own bodies, recognizing women’s agency [83] although women experience limited possibilities for negotiating condom use, given the current problematic ideologies of gender [84]. The various texts say little about women’s own agency, though, in accepting or rejecting the female condom. Several agencies recognize that female condoms are currently not universally accessible; the Ministry of Foreign Affairs of the Netherlands for instance states: “But the female condom is, for example, rarely available, and too expensive for most women” [59, p. 26].

According to UNFPA programming for female condoms has the goal “to develop strategies and programmes through which every sexually active person at risk of HIV or other sexually transmitted infections – regardless of age, marital status, gender, sexual orientation, economic status, cultural and religious beliefs or HIV status – has access to good quality condoms when and where he or she needs them; is motivated to use male or female condoms, as appropriate; and has the information and knowledge to use them consistently and correctly” [71, p. 6]. In its current on-going programmes, however, UNFPA like several other global actors normalize the use of the female condom for commercial sex workers rather than for women in general, as illustrated by the following quote: “More sex workers are using the female condom … probably because they are in a stronger position to negotiate than married women or single girls” [71, p. 16]. This is in contrast to male circumcision programmes which target men in general, without a restriction to any particular subcategory.

To sum up, the discursive power of global AIDS policy actors in framing the AIDS problem is located in defining gender from the perspective of women and girls, rather than men, and with a focus on reproductive health, rather than sexual health or sexual rights. This implies that women and girls are mainly portrayed as victims, rather than sexual agents. Women and girls are considered as homogenous groups, without any distinction for specific programmes or technologies. Policy makers target women as mothers in PMTCT programmes to prevent their new-borns from becoming HIV infected. Seldom, women are labelled as lesbians, and as a result they are excluded from AIDS policies. This denial of other sexual orientations of women illustrates the general disregard for women’s sexual agency. And this colours the prejudices about the female condom as a prevention technology. We argue that global AIDS policy actors contribute to re-enforcing the denial of women’s sexual agency. Other researchers also found that the existing gender stereotypes about sexual behaviours have hindered AIDS prevention [85,86].

## Discussion

The social environment represents social and sexual (risk) relations between all kinds of people. Running risks is considered as an expression of tension and of power relations in society. This view on risk means that the whole community is involved and everyone contributes one way or the other to risk-taking behaviour, not only high-risk groups defined as such by others [87]. This approach looks similar to the local approach towards AIDS prevention in Uganda, adopted at the start of the AIDS epidemic in the 1980s. We found a very different approach towards AIDS prevention among global AIDS policy actors who labelled specific categories of people at high risk. They do not consider AIDS as a threat to society as a whole, rather to specific categories of people. At the beginning of the AIDS epidemic in Uganda, AIDS prevention programmes were made accessible to all people, who could make their own free choice to use prevention methods, without any stigma. When the AIDS problem grew into a global issue, labelling people to target programmes and technologies became a common practice, excluding some categories and stigmatizing others. Global policies may be more effective if they avoid labelling but address the social environment in general and create space for local players to make sense of the AIDS problem. By targeting specific groups and labelling some and not others, they unwittingly “do gender” and so might contribute to less variety in and space for local responses to the AIDS epidemic, as also concluded by Ailio [88].

Normalizing female condom use for commercial sex workers rather than for other categories of women who are sexually active, implicitly links HIV to sexually immoral behaviour, thus stigmatizing HIV-positive women in their local communities. Once a woman is known to be HIV-positive, she is often seen as sexually immoral, which in fact is a negative interpretation of her sexual agency [39,40]. This might link to our finding that the attribution of sexual agency to women is limited to sex workers in global AIDS policy papers, which strengthens the association between HIV in women and sexually immoral behaviour. Men’s sexual agency is generally not negated, while women’s sexual agency is [89,90]. Women’s autonomy and power in sexual relations is often perceived “unfeminine” and threatening to men [23,91,92]. Female condoms, which may be seen to increase women’s power in sexual relations, can thus also be perceived as threatening. In general, women’s agency in accepting or resisting the female condom, and the effects this has on gender relations, need more profound study. A review of the literature on this issue, conducted in the context of another paper which is currentlt under review, shows, however, that women’s acceptance of the female condom and their readiness to use it in order to gain more control over their sexuality proves to be greater than that is generally assumed. This contrasts with the observed limited sexual agency attributed to women in AIDS prevention policies and the quasi-complete avoidance of framing the AIDS discourse in terms of sexual rights.

## Conclusions

The nature of the discursive power of global AIDS policy actors was the central theme of this article, with particular reference to the female condom. We found that global policy actors frame the AIDS epidemic mainly in the context of gender and reproductive health, rather than that of sexuality and sexual rights. Women and girls are often referred to, but more as victims than as specific target groups, and not as sexual agents. Female target groups are mostly mothers (focusing on their reproductive role) and commercial sex workers (focusing on their role to give men sexual pleasure), leaving out women in their own right, as sexual agents. Homosexual men (gays) are explicitly labelled to target AIDS prevention programmes, while homosexual women (lesbians) are not labelled at all. Among the prevention technologies, the condom is prioritized in the policy discourse. Although it remains gender unmarked, it is implicitly associated with men (men only), reinforcing the gender notion of sexual agency of men. It is almost taken for granted that (male) condoms need to be accessible for all men and not only specific categories of men. In contrast, the female condom is normalized for sex workers. None of the 16 policy papers analyzed make a serious attempt to insist on a programme of action to make female condoms universally accessible, that is: to all sexually active women. There is a deliberate effort to portray male circumcision as the norm for all men in sub-Saharan African countries that have high HIV prevalence rates, but without proper additional sexual education this may create extra risks for female partners. The gender-stereotyped AIDS policy discourse at the global level negates women’s agency in sexuality and her sexual rights. This in turn might have limited the scale-up of programmes that would make female condoms universally accessible.
